# A Comprehensive Behavioral Test Battery to Assess Learning and Memory in 129S6/Tg2576 Mice

**DOI:** 10.1371/journal.pone.0147733

**Published:** 2016-01-25

**Authors:** Andrea Wolf, Björn Bauer, Erin L. Abner, Tal Ashkenazy-Frolinger, Anika M. S. Hartz

**Affiliations:** 1 Department of Pharmacy Practice and Pharmaceutical Sciences, College of Pharmacy, University of Minnesota, Duluth, MN 55812, United States of America; 2 Department of Pharmaceutical Sciences, College of Pharmacy, University of Kentucky, Lexington, KY 40536, United States of America; 3 Sanders-Brown Center on Aging, University of Kentucky, Lexington, KY 40536, United States of America; 4 Department of Pharmacology and Nutritional Sciences, University of Kentucky, Lexington, KY 40536, United States of America; Texas Tech University Health Science Centers, UNITED STATES

## Abstract

Transgenic Tg2576 mice overexpressing human amyloid precursor protein (hAPP) are a widely used Alzheimer’s disease (AD) mouse model to evaluate treatment effects on amyloid beta (Aβ) pathology and cognition. Tg2576 mice on a B6;SJL background strain carry a recessive *rd1* mutation that leads to early retinal degeneration and visual impairment in homozygous carriers. This can impair performance in behavioral tests that rely on visual cues, and thus, affect study results. Therefore, B6;SJL/Tg2576 mice were systematically backcrossed with 129S6/SvEvTac mice resulting in 129S6/Tg2576 mice that lack the *rd1* mutation. 129S6/Tg2576 mice do not develop retinal degeneration but still show Aβ accumulation in the brain that is comparable to the original B6;SJL/Tg2576 mouse. However, comprehensive studies on cognitive decline in 129S6/Tg2576 mice are limited. In this study, we used two dementia mouse models on a 129S6 background—scopolamine-treated 129S6/SvEvTac mice (3–5 month-old) and transgenic 129S6/Tg2576 mice (11–13 month-old)–to establish a behavioral test battery for assessing learning and memory. The test battery consisted of five tests to evaluate different aspects of cognitive impairment: a Y-Maze forced alternation task, a novel object recognition test, the Morris water maze, the radial arm water maze, and a Y-maze spontaneous alternation task. We first established this behavioral test battery with the scopolamine-induced dementia model using 129S6/SvEvTac mice and then evaluated 129S6/Tg2576 mice using the same testing protocol. Both models showed distinctive patterns of cognitive impairment. Together, the non-invasive behavioral test battery presented here allows detecting cognitive impairment in scopolamine-treated 129S6/SvEvTac mice and in transgenic 129S6/Tg2576 mice. Due to the modular nature of this test battery, more behavioral tests, e.g. invasive assays to gain additional cognitive information, can easily be added.

## Introduction

Experts estimate that by 2050 up to 15 million Americans and more than 135 million people worldwide will suffer from Alzheimer’s disease (AD; [1]). AD is characterized by accumulation of amyloid beta (Aβ) and tau protein in the brain, which results in neurodegeneration followed by progressive cognitive decline [2–4]. However, although the pathological phenomena, as well as the key players of the disease—Aβ and tau protein—have been identified, a cure for AD is not available yet.

To study the effectiveness of new treatments for AD in animal models, current research combines molecular analysis with behavioral tests. This experimental approach was employed in 1996 by Ashe et al. using B6;SJL/Tg2576 mice, the first AD mouse model that exhibits both Aβ brain accumulation and cognitive deficits [5]. B6;SJL/Tg2576 mice overexpress the human amyloid precursor protein (hAPP) 695 isoform that harbors the Swedish double mutation found in some cases of familial AD [5]. At about 6 months of age, B6;SJL/Tg2576 mice have significantly increased brain levels of insoluble Aβ that aggregates to Aβ plaques at 7–8 months of age [6]. At 6–12 months of age, B6;SJL/Tg2576 mice develop cognitive impairment [7], which has been the focus of numerous studies [5, 8–10]. Although various tests have been used to characterize the behavioral/cognitive profile of B6;SJL/Tg2576 mice (reviewed in [11]), evaluating cognition in this AD model is challenging because one has to consider strain and model specific characteristics that can influence the results of cognitive testing.

In this regard, the B6;SJL background strain Tg2576 mice are bred on [5] carries a recessive *rd1* mutation which leads to early retinal degeneration so that homozygous *rd1* carriers lose almost all rod photoreceptor cells within the first 7 weeks after birth [12]. During this time, Tg2576 mice develop significant visual deficits, which impair their performance in cognitive tests that rely on visual cues and spatial orientation [13–17]. This problem was addressed by systematically backcrossing sixteen generations of the existing model with 129S6/SvEvTac mice, creating a Tg2576 mouse with a congenic 129S6 background [18]. Tg2576 mice with the 129S6 background lack the *rd1* mutation, and therefore, do not develop retinal degeneration, but still show Aβ accumulation in the brain that is comparable to the original Tg2576 mouse model [18]. Thus, using 129S6/Tg2576 mice when testing behavior eliminates potential problems arising from blindness observed with B6;SJL/Tg2576 mice. However, behavioral studies have mainly been conducted with B6;SJL/Tg2576 mice and a comprehensive cognition analysis of 129S6/Tg2576 mice is lacking.

Cognitive decline in 129S6/Tg2576 mice has been documented by few behavioral studies [18–20]. The most comprehensive study has been conducted by Ohta et al. [20], who assessed behavior and cognition of 129S6/Tg2576 mice at 3, 6 and 12 months of age using various tests. However, data from studies using a combination of behavioral tests to characterize 129S6/Tg2576 mice are limited. Such information is critical to understand changes—both, molecular and behavioral—resulting from testing new therapeutic strategies that utilize the 129S6/Tg2576 mouse AD model.

In the present study, we established a comprehensive test battery to assess learning and memory in two different dementia mouse models on a 129S6 background. The test battery consisted of five tests that were performed in the order: forced alternation (Y-maze), novel object recognition, Morris water maze, radial arm water maze, and spontaneous alternation (Y-maze). We first established this behavioral test battery with the scopolamine-induced dementia model using mice on a 129S6 background, and then evaluated 11–13 month-old 129S6/Tg2576 mice with the same test battery following the same protocols.

## Materials and Methods

### Chemicals and Equipment

Scopolamine-HBr was purchased from Sigma-Aldrich (St Louis, MO) and saline (0.9% NaCl) was from Hospira (Lake Forest, IL). Scopolamine-HBr was dissolved in saline and freshly prepared each day animals were dosed. The Morris water maze pool was purchased from Harvard Apparatus (Holliston, MA); all other testing equipment was purchased from Stoelting Co. (Wood Dale, IL).

### Animals

All animal experiments were approved by the Institutional Animal Care and Use Committee of the University of Minnesota (protocol# 1110A05865; PI: A. Hartz) and carried out in accordance with AAALAC regulations, the US Department of Agriculture Animal Welfare Act, and the Guide for the Care and Use of Laboratory Animals of the NIH.

Male 129S6/SvEvTac mice, male Tg2576 mice (129S6.Cg-Tg(APPSWE)2576Kha N20+?) and the corresponding male wild type (WT) mice were all from Taconic (Germantown, NY). Littermate control mice (WT) and Tg2576 mice were obtained from the same 129S6 colony. The APPSWE mouse was developed by Dr. Karen Hsiao at the University of Minnesota in collaboration with the Mayo Clinic (Rochester, MN). The APPSWE model was created by microinjecting the human APP695 gene containing the double mutation K670N and M671L into B6SJLF2 zygotes using a hamster prion protein cosmid vector. The resultant mice from the Founder Line 2576 were backcrossed sixteen generations (N16) to 129S6 mice. Taconic received the stock in September 2003. Mice were derived by embryonic transfer and are maintained by backcrossing hemizygous male mice with 129S6/SvEvTac female mice. For the scopolamine-induced dementia model, male 129S6/SvEvTac mice were randomly selected for i.p. injection (5 ml/kg) with scopolamine-HBr (10 mg/kg, i.p., n = 10) or vehicle (0.9% NaCl, n = 10, control group). Mice were single-housed due to the aggressive behavior of male Tg2576 mice and kept on a 12 hour light/dark cycle with food and water *ad libitum*. For behavioral tests with scopolamine-injected mice and vehicle-treated control mice, animals were allowed to adapt to the new environment for 10 weeks and were tested at 3–5 months of age. 129S6/Tg2576 mice (n = 20) and corresponding WT mice (n = 20) were obtained at 12 weeks of age and tested at 11–13 months of age. All tests were conducted in the light phase, the order of behavioral tests was the same for each mouse. One mouse died of an unknown cause between the radial arm water maze test and the spontaneous alternation test.

### Behavioral Test Battery

**Fig 1:**
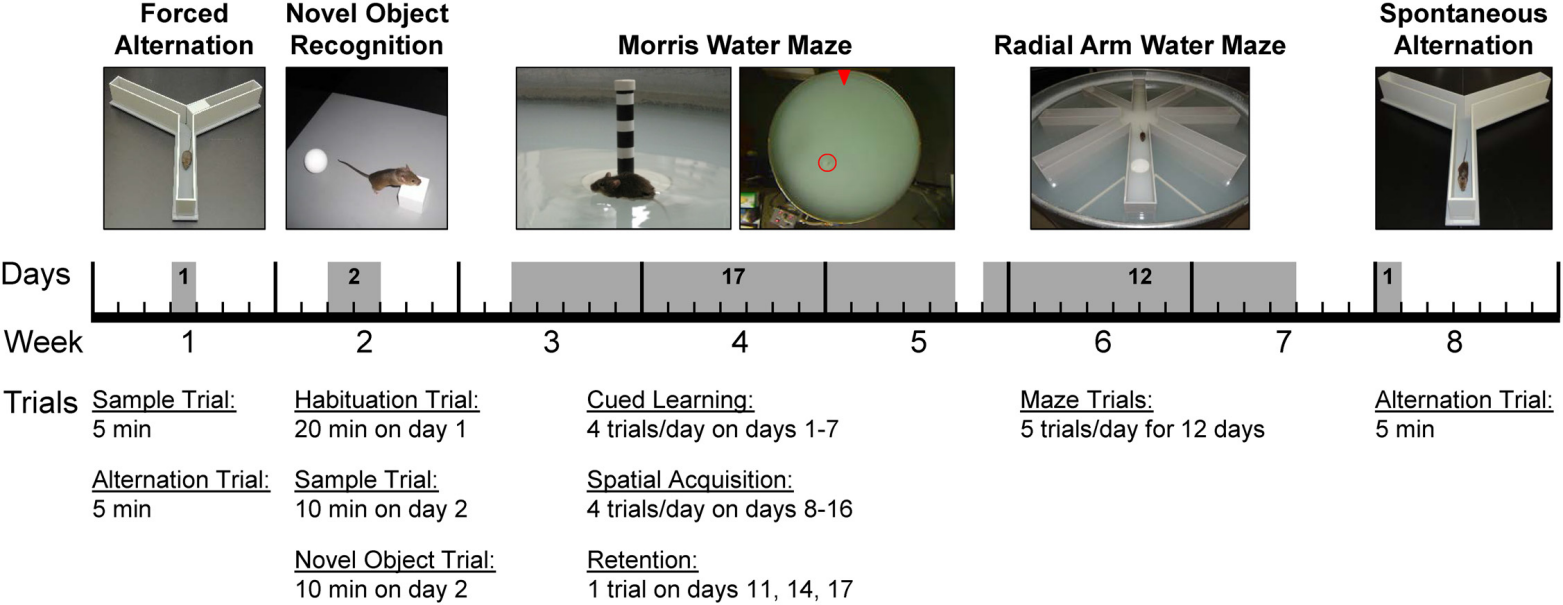
Behavioral Test Battery. Mice were tested in a behavioral test battery consisting of (top panel, from left to right): forced alternation (Y-maze), novel object recognition, Morris water maze, radial arm water maze, and spontaneous alternation (Y-maze) over a period of 8 weeks. Top panel: Behavioral tests conducted in the study. Middle panel: Timeline of tests. Bottom panel: Trials conducted per behavioral test. Tests were conducted in the order of increasing invasiveness. The spontaneous alternation test was performed last to maximize the interval to the forced alternation test, which utilized the same maze.

The behavioral test battery consisted of five memory tests performed in the following order: Y-maze forced alternation, novel object recognition, Morris water maze, radial arm water maze and Y-maze spontaneous alternation (Fig 1). Tests were conducted in the order of increasing invasiveness, with the exception of the Y-maze spontaneous alternation test. This test was conducted at the end of the test battery to minimize interference with the forced alternation test at the beginning of the study. Mice had multiple days of resting time between tests to decrease carryover effects from prior tests. The order of tests in which mice were tested was the same for each mouse; each mouse was tested once per test. Black and white cues were placed at the walls around the testing area for all tests except the novel object recognition trials. Cues were changed after the forced alternation test and remained the same for the remaining tasks. All test trials were video-recorded, tracked, and analyzed with ANY-maze^™^ tracking software (version 499g Beta). Locomotor activity data for each test are summarized in Table 1. Mice were habituated to the testing room for 30 min at the beginning of each test day. All tests in the battery were conducted by the same experimenter. During the test trials, the experimenter was separated from the testing area by a curtain. All experiments comparing 129S6/Tg2576 to wild type mice were blinded. Memory tests are described in the order the tests were conducted.

**Table 1:** Locomotor Activity. A) Locomotor activity for control and scopolamine-treated mice, B) locomotor activity for WT and Tg2576 mice. Values are mean ± SEM; Student’s *t*-test was used to calculate *p* values.

**Table 1A**	**Ctrl**	**n**	**SCP**	**n**	***p* (two tailed)**	**df**
FA (arm entries, T1)	22.7 ± 1.7	9	35.6 ± 3.3	9	0.003	16
FA (arm entries, T2)	5.8 ± 0.3	9	7.3 ± 0.6	9	0.041	16
NOR distance habit trial [m]	24.7 ± 1.9	9	24.6 ± 2.4	8	0.989	15
MWM distance Day 11 [m]	11.2 ± 0.5	10	11.4 ± 5.4	10	0.726	18
MWM distance Day 14 [m]	10.9 ± 0.4	10	11.8 ± 0.4	10	0.118	18
MWM distance Day 17 [m]	11.1 ± 0.3	10	10.9 ± 0.4	10	0.763	18
SA (Total Arm Entries)	13.7 ± 1.2	9	21.6 ± 2.1	9	0.006	17
**Table 1B**	**WT**	**n**	**Tg2576**	**n**	***p* (two tailed)**	**df**
FA (arm entries, T1)	24.3 ± 0.9	18	24.1 ± 1.4	18	0.868	34
FA (arm entries, T2)	7.3 ± 0.3	18	6.5 ± 0.6	18	0.234	34
NOR distance habit trial [m]	37.1 ± 2.8	20	37.9 ± 4.2	19	0.871	37
MWM distance Day 11 [m]	12.5 ± 0.4	18	12.5 ± 0.4	19	0.988	35
MWM distance Day 14 [m]	11.5 ± 0.4	18	11.6 ± 0.2	19	0.848	35
MWM distance Day 17 [m]	11.2 ± 0.3	18	11.7 ± 0.3	19	0.340	35
SA (Total Arm Entries)	17.6 ± 1.3	19	16.1 ± 1.5	14	0.442	31

### Forced Alternation Test (Y-Maze)

Forced alternation tests were conducted using a symmetrical Y-maze made of a grey steel bottom plate with grey Perspex^®^ walls (Stoelting Co, Wood Dale, IL). Each arm of the Y-maze was 35 cm long, 5 cm wide, and 10 cm high, and the wall at the end of each arm was marked with a different black and white pattern. To reduce anxiety in the animals, light in the testing area was dimmed to 30 ± 5 lux.

The protocol for the forced alternation test was modified from Melnikova et al. [21]. Mice were handled for three days before testing. The test consisted of a 5 min sample trial (T1) followed by a 5 min retrieval trial (T2). For the scopolamine experiment, mice were dosed with scopolamine or vehicle 30 min before T1. In T1, the mouse was placed into the end of the start arm, facing the wall and away from the center. The mouse was then allowed to explore two arms of the Y-maze, while entry into the third arm was blocked. After the sample trial, the mouse was returned to its home cage for a 30 min inter-trial interval. In T2, the block in arm 3 was removed, the mouse was again placed into the start arm, and then allowed to access all three arms of the maze. If a mouse climbed on the maze wall, it was immediately returned into the abandoned maze arm. After each animal and between T1 and T2, the maze was wiped with a Quatricide^®^ dilution to prevent odor cues. An arm entry was recorded when 85% of a mouse’s body entered the arm. Time in Novel Arm [%] was defined as the time spent in the novel arm divided by the time spent in all arms during the first minute of the retrieval trial T2. Forced Alternation [%] was defined as the percent of mice entering first the novel arm during T2 [22]. Mice with less than three arm entries in the first minute of T2 were excluded from the analysis.

### Novel Object Recognition Test

Novel object recognition trials were conducted in square test boxes (40x40x35 cm) with even lighting conditions (30 ± 5 lux). Each test box consisted of a white Perspex^®^ bottom plate surrounded by black Perspex^®^ walls and a camera positioned centrally above each box. A white-painted wooden cube and a white-painted wooden sphere were used as objects (Fig 1). Prior to the experiments, both objects were tested with a separate cohort of mice to exclude that mice showed a preference for either object (data not shown). The use of the sample object and the novel object as well as the placement of the novel object followed a counterbalanced design between trials to control for order and location effects.

Prior to testing, mice were handled for 2 days. On day 1 of the tests, we conducted a habituation trial where mice were allowed to explore the empty test box for 20 min. Twenty-four hours after the habituation trial (day 2), a sample trial was conducted. For the scopolamine experiments, mice were dosed 30 min before the sample trial. In the sample trial, mice were placed into the empty test box always in front of the south wall facing away from the objects. After a 5 min customization phase, two equal sample objects (e.g., two cubes) were introduced into the box. The mouse was allowed to explore the objects for 10 min before it was returned to its home cage. After 60 min, the novel object trial was conducted by placing the mouse into the empty test box again. After an initial 5 min customization phase, one sample object and one unfamiliar object (a cube and a sphere) were introduced and object interaction was recorded for 10 min. Object interaction was defined as an event where a mouse’s head was within 2 cm of the object and directed towards the object. Sitting or leaning on the object was not considered object exploration [23, 24]. Object Interaction [%] for the sample object was calculated as (sample object interaction x 100) / (sample object interaction + novel object interaction). Object interaction [%] for the novel object was calculated as (novel object interaction x 100) / (sample object interaction + novel object interaction) [25]. After each trial, the objects and boxes were cleaned with a Quatricide^®^ dilution to eliminate odor cues. Mice with less than 7 s of total object interaction in either trial were excluded from the analysis [26].

### Morris Water Maze Test

The protocol for Morris water maze tests was adapted from Vorhees et al. [27] with the following modifications. We used a circular pool (150 cm diameter) filled with water (26 ± 1°C; rim height (distance water surface to wall rim): 10 cm) that was made opaque with non-toxic tempera paint. A circular rescue platform (diameter: 11 cm; distance between platform center point and pool wall: 27 cm) was submerged 1–1.5 cm below the water surface and the testing area was illuminated with indirect lighting (50 ± 10 lux) to avoid reflections. To monitor animals during trials, a camera was mounted to the ceiling centrally above the pool. The water maze was surrounded by black-and-white extra-maze cues on the walls of the room.

Repeated episodes of excessive floating (>10 s/trial in ≥ 25% of trials) was rare and found in only 3 (two WT mice, one Tg2576 mouse) out of 57 mice during the entire study. These mice were excluded from the analysis [28]. Floating was empirically determined as swimming with a speed below 4 cm/second. One 129S6/Tg2576 mouse died of an unknown cause during the cued learning phase and was excluded from the analysis. For the scopolamine study, mice were dosed 30–40 min prior to the first trial of each day.

#### Cued Learning Phase

Prior to testing, mice were handled for two days. For the cued learning trials, the circular rescue platform was tagged with a black and white pole extending 12 cm above the water surface. Each mouse was gently lowered into the water, facing the wall of the pool, and then allowed to swim freely until it found the platform. Once the platform was found, mice were allowed to sit on the platform for 5 s before being dried with a towel and returned to a heated drying cage. Mice that did not find the platform within 1 minute were guided to the platform and assigned a latency of 60 s. Over the course of seven days, each mouse completed four trials per day (inter-trial interval: 20–30 min). On day 1, each mouse was placed on the platform for 15 s immediately before the first trial. According to Vorhees and Williams, the starting and the platform locations between trials were varied semi-randomly [27].

#### Spatial Acquisition Phase

Spatial acquisition trials started one day after the cued learning phase. The pole was removed from the submerged rescue platform to make it invisible to the mice. In order to locate the escape platform, mice now had to navigate through the pool using extra-maze cues. The platform location remained the same for all trials, whereas the starting location varied between trials. Mice had 60 s to find the rescue platform, after which they were guided there. Each mouse performed four trials per day over nine days. On day 1 of the spatial acquisition trials, mice were placed on the platform for 15 s immediately before the first trial.

#### Retention Trials

At the beginning of the 4^th^, 7^th^ and 10^th^ day of the spatial acquisition phase (i.e., after every 12^th^ acquisition trial), a retention trial was conducted. For these trials, the rescue platform was removed from the pool and the mouse was allowed to swim for 60 s.

For the cued learning and the spatial acquisition trials, the mean latency to reach the platform was calculated for each test day [27]. For the retention trials, the percent of the time each mouse spent in the target quadrant (the NE quadrant) was analyzed, as well as the percent time spent in an annulus (40 cm diameter) surrounding the platform center point.

### Radial Arm Water Maze Test

The protocol for the radial arm water maze tests was adapted from Savonenko et al. [29] with the following modifications: the radial arm water maze test was conducted in the same pool as the Morris water maze test. A radial maze insert with 8 arms (arm length: 50 cm, width: 10 cm, height: 20 cm, rim height: 6 cm) was placed in the center of the pool. A circular platform (diameter: 9 cm) was placed in one of the arms, submerged by 1–1.5 cm under the water surface. The water was made opaque by the addition of non-toxic tempera paint and water temperature was held constant at 26 ± 1°C. The same extra-maze cues and lighting conditions were used as for the Morris water maze trials.

The radial arm water maze trials started one day after the last Morris water maze retention trial. The rescue platform was moved after each test day so that mice had to learn and remember the platform location every day. Facing away from the center, the mouse was placed into the water at the end of the start arm, from where it swam freely until it located the rescue platform. If the mouse failed to locate the platform within 60 s, it was gently guided there by the experimenter. Each mouse conducted five trials per day over twelve days, with the start arm varying between trials. The first four trials of each day, in which the mouse learned the platform location, were conducted with a 3–10 min inter-trial interval. A fifth trial, the retention trial, was conducted 30 min after trial 4. On day 1, mice were put on the platform for 15 s immediately before the first trial. During the test trials none of the mice tested showed extensive floating (> 10 s/trial). For the scopolamine experiments, mice were dosed 30–40 min before the first trial of each day.

For each trial, the errors made by the mouse in finding the platform were added up until it reached the platform. An error was charged each time the mouse entered an arm different from the target arm, or if it failed to enter an arm for 15 s. Entering the target arm without locating the platform was not counted as an error. The test was conducted over twelve days; the last three days were averaged for analysis. To determine long-term memory, entries into the target arm of the previous day’s first trial were also analyzed for days 2–12 [29].

### Spontaneous Alternation Test (Y-Maze)

Spontaneous alternation tests were conducted in the same Y-maze as described above. For this test, the Y-maze was rotated by 45° and the distal cues differed between the forced and the spontaneous alternation test. This test consisted of a single 5 min trial, in which the mouse was allowed to explore all three arms of the Y-maze. If a mouse climbed on the maze walls, it was immediately returned to the abandoned arm. The start arm was varied between animals to avoid placement bias. For the scopolamine experiment, mice were dosed with scopolamine or vehicle 30–40 min before the experiment. Spontaneous Alternation [%] was defined as consecutive entries in 3 different arms (ABC), divided by the number of possible alternations (total arm entries minus 2; [30]). Re-entries into the same arm were rated as separate entries, which resulted in a 22.2% chance level for continuous alternation [31]. Mice with less than 8 arm entries during the 5-min trial were excluded from the analysis [18].

### Statistics

**Fig 2:**
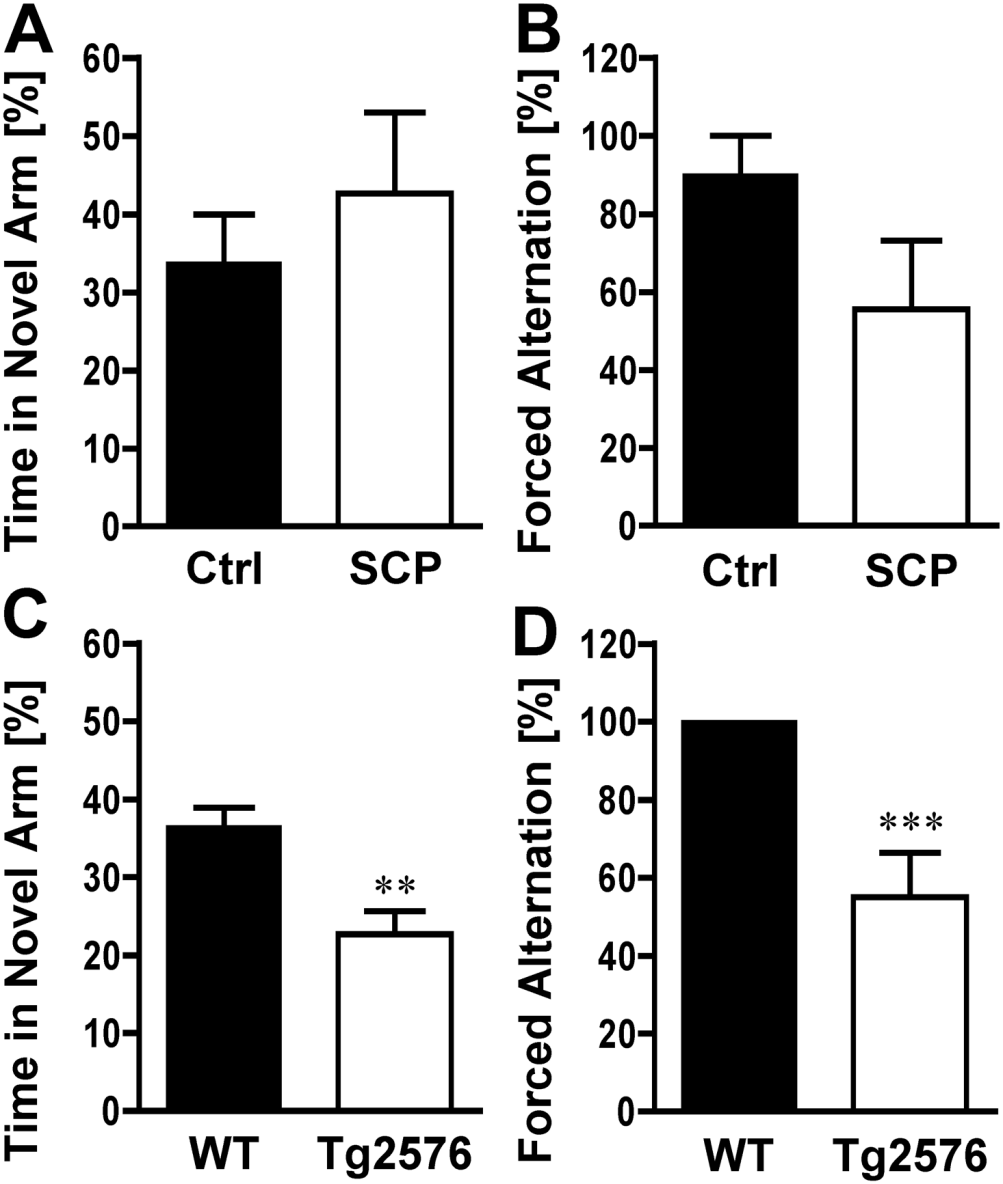
Forced Alternation (Y-Maze). **A)** Scopolamine-HBr (10 mg/kg) or saline was administered by i.p. injection 30 min before the sample trial. A retrieval trial was conducted 30 min after the sample trial. The time spent in the novel arm during the first minute of the retrieval trial was analyzed. For both treatment groups (saline control: Ctrl; scopolamine: SCP), time spent in the novel arm was about 33%, with no statistically significant difference between groups (n = 9/group). **B)** In the second arm entry of the retrieval trial, saline-injected control mice entered the novel arm less often than scopolamine-treated mice (SCP). **C)** 129S6/Tg2576 mice (11–13 months) spent significantly less time in the novel arm during the retrieval trial and **D)** entered significantly less often into the novel arm (n = 19-20/group). The control has no error bar because all mice (100%) first went into the novel arm. Mice with less than 3 arm entries in the first minute were excluded from the analysis. Data are mean ± SEM; **p < 0.01; ***p < 0.001 (Student’s *t*-test for Fig 2A and 2C; Chi-squared test for Fig 2B and 2D).

**Fig 3:**
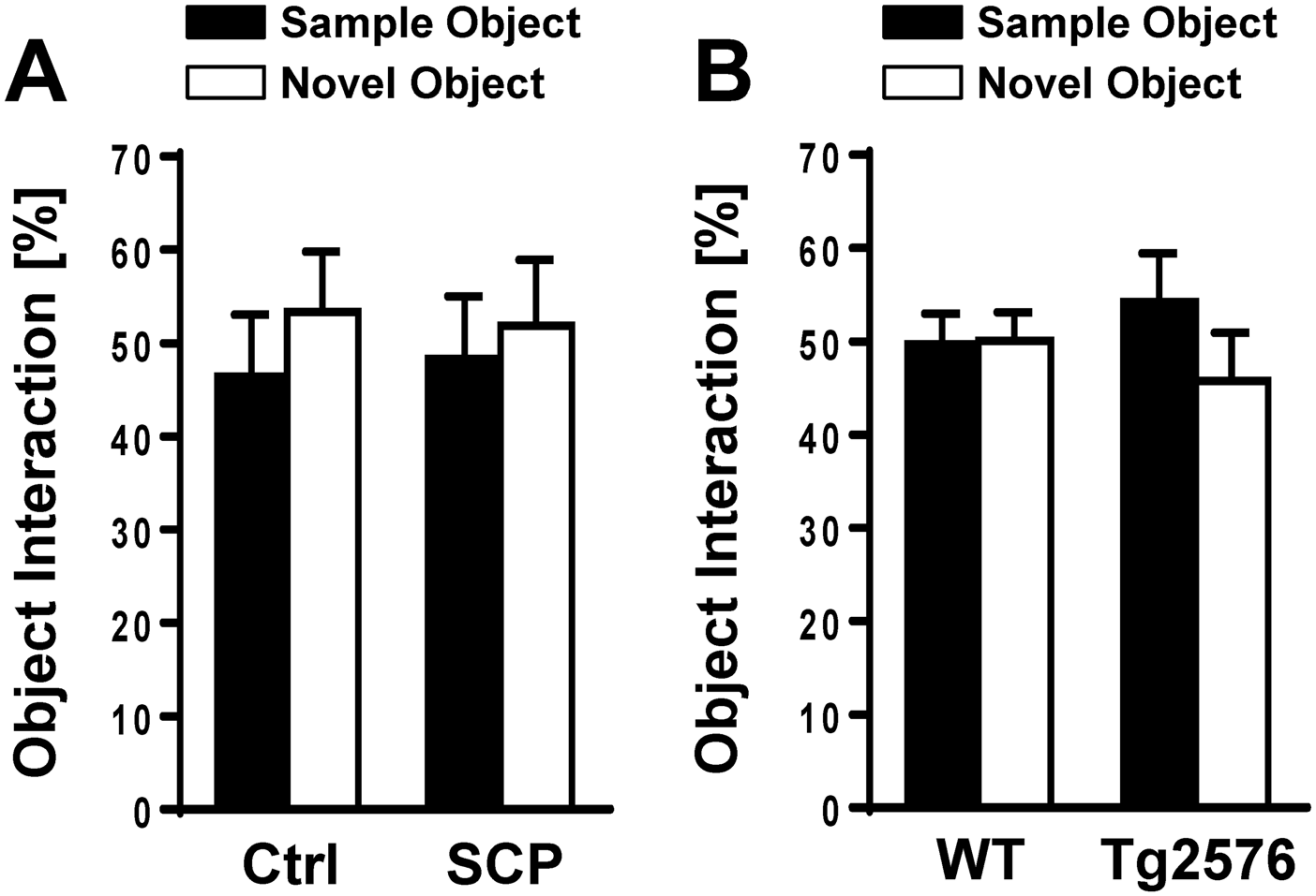
Novel Object Recognition. **A)** Both control (Ctrl) and scopolamine-treated (SCP) mice showed no preference for the novel object over the familiar object (n = 8-9/group), but control mice showed a trend to interact more with the novel object (*p* = 0.466). **B)** Tg2576 mice showed a trend to interact less with the novel object (*p* = 0.257; n = 18-19/group). Mice with less than 7 sec of object interaction in either trial were excluded from the analysis. Data are mean ± SEM; Student’s *t*-test was used to calculate *p* values.

**Fig 4:**
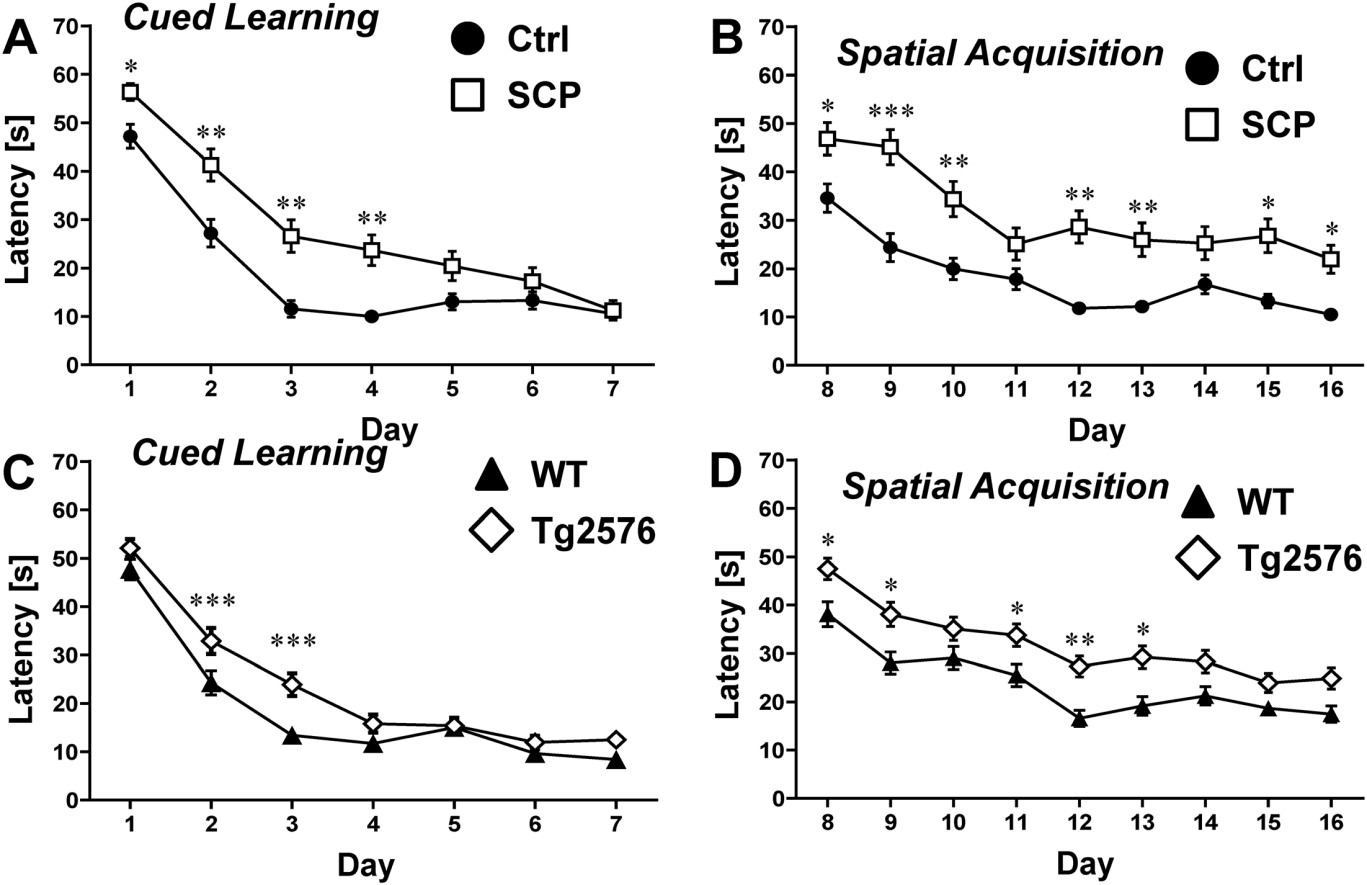
Morris Water Maze: Cued Learning and Spatial Acquisition. **A)** In the first four days of the cued learning trial, the latency time to locate the visible platform was significantly longer for scopolamine-treated mice (SCP) compared to untreated control mice (Ctrl; n = 10/group). (**B)** The latency of scopolamine-treated mice was significantly longer compared to control mice in the spatial acquisition trials. **C)** Compared to WT mice, transgenic 129S6/Tg2576 mice had consistently higher latency times to find the tagged platform in the cued learning phase (n = 18-19/group). **D)** The latency time to find the hidden platform was higher for 129S6/Tg2576 mice than for WT mice, but this effect was only statistically significant for days 8, 9, 11, 12 and 13. Mice that showed repeated episodes of extensive floating (> 10 s/trial; > 25% of trials) were excluded from the analysis of the entire experiment. Data are mean ± SEM; *p < 0.05; **p < 0.01; **p < 0.001 (repeated measures ANOVA).

**Fig 5:**
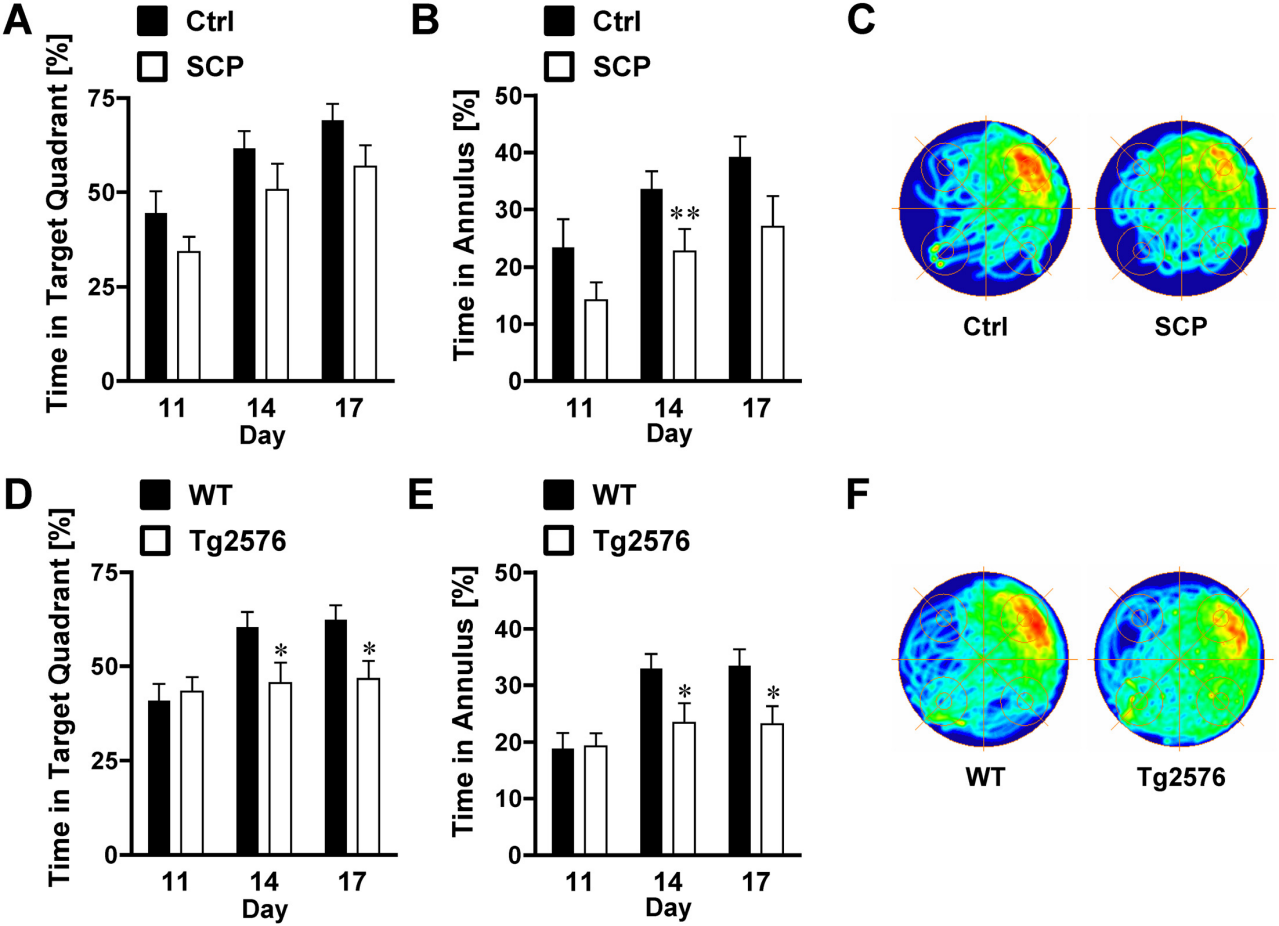
Morris Water Maze: Retention of Spatial Reference Memory. **A)** Compared to control mice (Ctrl), scopolamine-treated mice (SCP) spent less time in the target quadrant during the retention trial on days 14 and 17 and **B)** in the 40 cm annulus around the platform center on day 14 (n = 10/group). **C)** The group occupancy plot from the last retention trial (day 17) shows a similar search pattern for both groups. Red and yellow regions represent areas of high occupancy; green and blue represent areas of low occupancy. **D)** On days 14 and 17 of the retention trials, 129S6/Tg2576 mice spent less time in the target quadrant compared to WT control mice (statistically not significant). **E)** 129S6/Tg2576 mice spent significantly less time in the 40 cm platform annulus compared to age-matched WT control mice (n = 18-19/group) on days 14 and 17 of the retention trials. **F)** Occupancy plots obtained from WT and transgenic mice in the last retention trial (day 17) exhibited a similar search pattern for both groups. Mice that showed repeated episodes of extensive floating (> 10 s/trial; >25% of trials) were excluded from analysis for the entire experiment. Data are mean ± SEM; *p < 0.05; **p < 0.01 (Student’s *t*-test).

**Fig 6:**
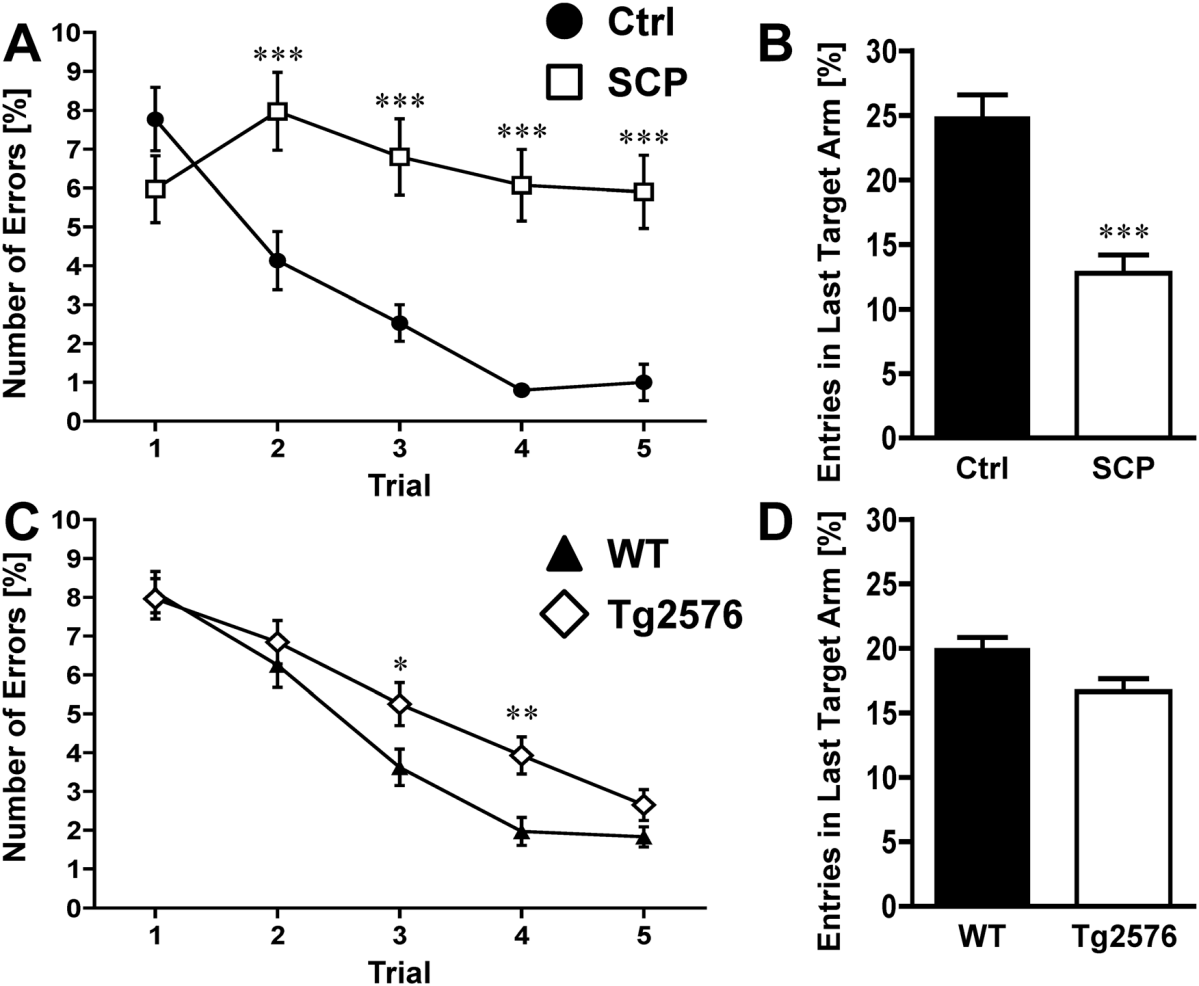
Radial Arm Water Maze. **A)** Scopolamine-treated mice (SCP) made more errors at learning (trials 1–4) and remembering (trial 5) the hidden platform location than control mice (Ctrl) did (n = 9-10/group). **B)** Long-term (24 h) memory of mice was evaluated by averaging the entries into the previous target arm during the first trial of days 2–12. SCP mice entered into the last target arm significantly less than control mice. **C)** 129S6/Tg2576 mice made significantly more errors during the learning phase (trials 1–4) of the radial arm water maze test compared to WT control mice. **D)** The number of entries into the last target arm were not different between 129S6/Tg2576 mice and WT control mice (n = 19-20/group). Data are mean ± SEM; 6A and 6C: *p < 0.05; **p < 0.01; ***p < 0.001 (repeated measures ANOVA); 6B and 6D: ***p < 0.001 (repeated measures ANOVA was used for Fig 6A and 6C; Student’s t-test was used to calculated p values for Fig 6B and 6D).

**Fig 7:**
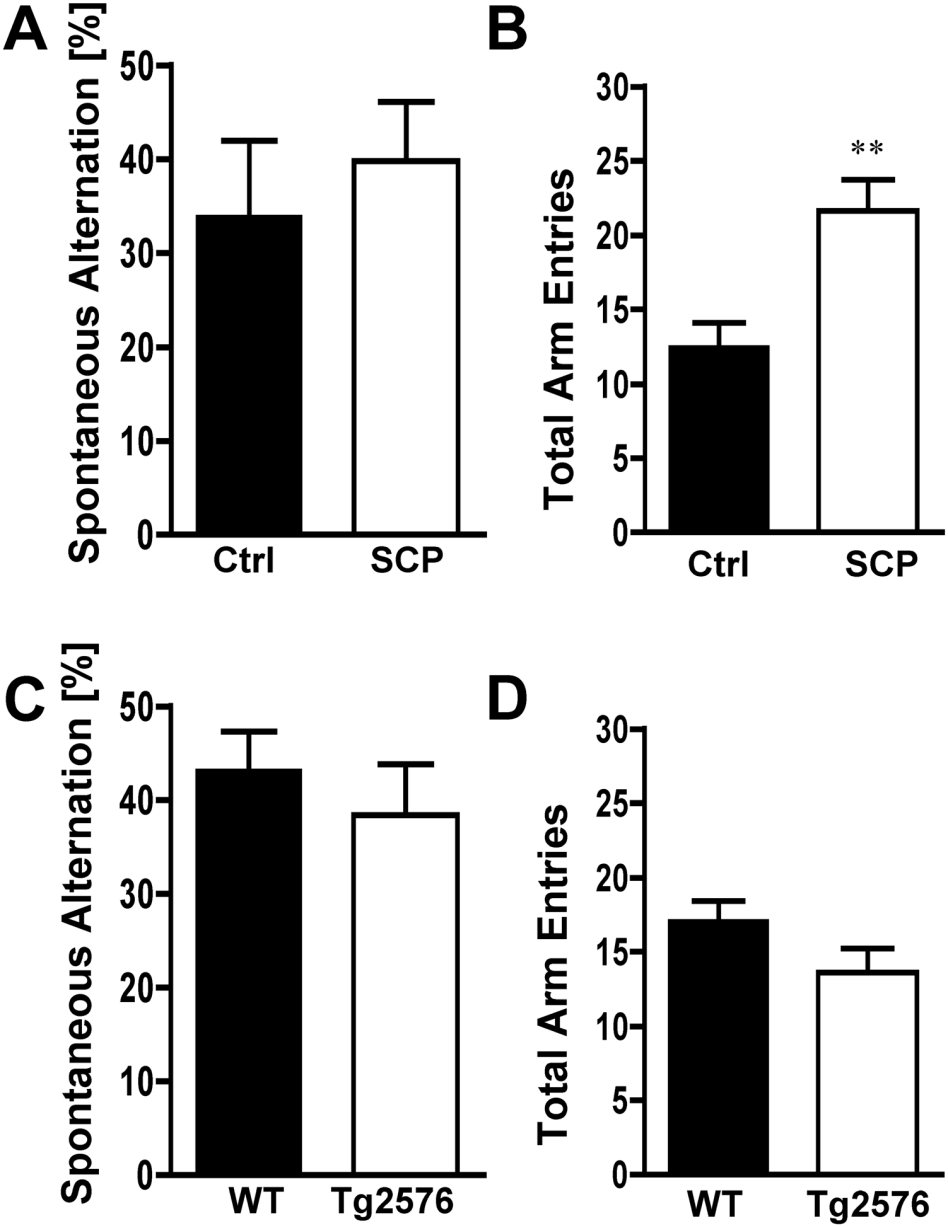
Spontaneous Alternation (Y-Maze). **A)** Scopolamine-injected mice (SCP) and control (Ctrl) mice showed the same level of spontaneous alternation (n = 9-10/group). **B)** Scopolamine-treated mice had significantly more arm entries than control mice. **C)** 129S6/Tg2576 mice and WT mice performed showed the same level of spontaneous alternation (n = 16-19/group). **D)** The number of total arm entries between WT and 129S6/Tg2576 mice was not statistically different. Mice with less than 8 arm entries were excluded from the alternation analysis. Data are mean ± SEM; **p < 0.01 (Student’s *t*-test).

Data are presented as mean ± SEM. A two-tailed unpaired Student’s *t*-test (assuming equal variance based on F-test; df = n_1_ + n_2_−2) was used to evaluate differences between groups in Table 1 and Figs 2A, 2C and 3 using ANY-maze^™^ software (version 499g Beta). The Chi-squared test was used to calculate statistical differences for Fig 2B and 2D. Using SAS statistical software (version 9.3; df_TOTAL_ = n– 1), statistical differences were calculated using repeated measures ANOVA for Fig 4. A two-tailed unpaired Student’s *t*-test as described above was also used to evaluate differences between groups in Figs 5, 6B, 6D, and 7 using ANY-maze^™^ software (version 499g Beta). Repeated measures ANOVA was also performed for Fig 6A and 6C. Differences were considered statistically significant when *p* < 0.05.

## Results

The behavior of mice can vary significantly by lineage, resulting in different cognitive outcomes for each strain [32–36]. Therefore, to fully assess learning and memory of a disease mouse model, it is critical to characterize the cognitive profile of the corresponding background mouse strain. The 129S6 mouse is the background strain for transgenic 129S6/Tg2576 AD mice overexpressing hAPP and lacking the *Pde6b rd1* retinal degeneration mutation. Compared to the original Tg2576 mice that bear the *Pde6b rdl* mutation, 129S6/Tg2576 mice are a newer AD model for which behavioral data are limited. To examine the cognitive profile of Tg2576 mice on a 129S6 background, we established a behavioral test battery consisting of forced alternation (Y-maze), novel object recognition, Morris water maze, radial arm water maze, and spontaneous alternation (Y-maze). We established this test battery with 3-5-month old scopolamine-induced dementia 129S6/SvEvTac mice and used the battery to determine the cognition of 11-13-month old 129S6/Tg2576 AD mice. Fig 1 shows the experimental design of the study. In the following, our results are described in the order the tests were conducted.

### Forced Alternation (Y-Maze)

Alternation tasks measure the disposition of rodents to explore new environments and are used to evaluate working memory and exploratory behavior [23, 37]. Fig 2A shows the percent time spent in the novel arm for saline-injected control mice and scopolamine-dementia mice. For both groups, the percent time spent in the novel arm was between 30–40%, with no significant difference between the groups (33.7% vs. 42.7%; *p = 0*.*469*). Analysis of the second arm entry in the retrieval trial revealed that control mice showed a higher level of alternation compared to scopolamine-injected mice (90% vs. 55.6%), although this difference was not statistically significant (Fig 2B, *p = 0*.*098*).

When we compared the behavior of 11-13-month-old 129S6/Tg2576 mice with that of age-matched WT mice, the percent time spent in the novel arm was significantly higher for WT mice compared to 129S6/Tg2576 mice (36.4% ± 2.5 vs. 22.7% ± 2.9; *p = 0*.*001*, Fig 2C). Consistent with this, alternation for WT mice was significantly (*p = 0*.*0001*) higher compared to that for transgenic 129S6/Tg2576 mice (100% ± 0 vs. 55.5% ± 11.4; Fig 2D).

### Novel Object Recognition

The novel object recognition test is used to assess recognition memory in mice and is based on the inherent tendency of rodents to explore a novel object longer than a familiar one [38]. Fig 3 shows that the percentage of object interaction of all four groups of mice tested ranged from 45.8% to 54.2%. Control mice (Fig 3A) showed a trend to interact more with the novel object (not significant, *p* = 0.466) and Tg2576 mice showed a trend to interact less with the novel object (not significant, *p* = 0.257). In both experiments, there were trends, but no significant differences between sample and novel objects were observed, suggesting that the mice had difficulties to distinguish between the novel and the familiar object.

### Morris Water Maze

To evaluate spatial learning and memory, mice were tested in a Morris water maze. Scopolamine-treated mice were impaired in all three phases of the experiments: 1. *Cued learning phase* (platform tagged with black/white pole), 2. *Spatial acquisition phase* (platform submerged) and 3. *Retention phase* (platform removed).

During the first four days of the *cued learning phase*, escape latencies were significantly different between saline and scopolamine-treated mice (Fig 4A). On day 1, the average escape latency of saline-treated mice was 47.2 ± 2.5 s and 56.4 ± 1.8 s for scopolamine-treated mice (*p* < 0.05). On day 2, latencies of scopolamine-treated mice were in average 41.3 ± 3.3 s, and thus, significantly higher compared to latencies of control mice (27.2 ± 2.8 s; *p* < 0.01). On days 3 and 4, latencies of scopolamine-treated mice were 2-fold higher compared to control mice (26.6 ± 3.3 s and 23.7 ± 3.1 s vs. 11.6 ± 1.7 s and 10 ± 1.1 s, respectively). Escape latencies on days 5–7 were not significantly different between scopolamine-treated mice and control mice (day 5: 13.0 ± 1.7 s vs. 20.5 ± 3 s; day 6: 13.3 ± 1.8 s vs. 17.3 ± 2.8 s; day 7: 10.6 ± 1.1 s vs. 11.3 ± 2 s). Fig 4B shows that escape latencies to find the hidden platform are significantly increased for scopolamine-treated mice compared to control mice during the entire *spatial acquisition phase* (days 8–16). On the first day of the spatial acquisition phase (day 8 of the entire Morris water maze test), the average latency of control mice was 34.6 ±2.9 s compared to 46.8 ± 3.3 s for scopolamine-treated mice (*p* < 0.05). Escape latencies for scopolamine-treated mice were in average 1.4–2.4 fold higher per time point compared to control mice and remained 2-fold higher at the final day (day 16) of the test (10.5 ± 0.9 s vs. 22.0 ± 2.9 s).

In retention trials, control mice spent about 21% more time in the target quadrant (5A) with increases in the percent time spent in the target quadrant on day 14 (61.6 ± 4.6% vs. 50.7 ± 6.9%; *p* = 0.206) and day 17 (69.1 ± 4.3% vs. 56.9 ± 5.6%; *p* = 0.103). Moreover, control mice spent 65–68% more time in the platform annulus (Fig 5B) than scopolamine-treated mice, with a significant increase in annulus time on day 14 (33.6 ± 2.8 s vs. 22.7 ± 3.8 s; *p* = 0.004) and day 17 (39.2 ± 3.6 s vs. 27 ± 5.3 s; *p* = 0.075). Overall, individual swim paths (S1 Fig) and density plots for the grouped data shown in Fig 5C indicate that search patterns of saline-treated mice are more precise than those of scopolamine-treated mice.

Similar to the scopolamine-induced dementia mice, 129S6/Tg2576 mice were impaired during all stages of the experiment, although the differences to their controls were less prominent. On day 1 of the *cued learning phase*, WT and transgenic 129S6/Tg2576 mice displayed escape latencies of 47.7 ± 2.1 s and 52.1 ± 1.8 s, respectively (Fig 4C). Escape latencies were significantly different for control mice vs. 129S6/Tg2576 mice on day 2 (24.3 ± 2.4 s vs. 32.9 ± 2.7 s; *p* < 0.05) and day 3 (13.4 ± 1.2 vs. 23.9 ± 2.3; *p* = < 0.01). On the last day of this phase (day 7), WT mice showed a latency of 8.4 ± 0.8 s and transgenic mice showed one of 12.5 ± 1.2 s.

During the *spatial acquisition phase*, latencies of 129S6/Tg2576 mice were in average about 37% higher than those of WT mice (Fig 4D). On the first day of the spatial acquisition phase (day 8 of the entire Morris water maze test), the escape latency for WT mice was 38.1 ± 2.6 s. Over the course of 9 days, this number dropped to 17.5 ± 1.7 s. In contrast, transgenic animals had an escape latency of 47.5 ± 2.2 s on the first day (day 8) and showed a latency of 24.8 ± 2.2 s on the last day (day 16), which is 42% higher than the latency for WT mice. During the *retention trials*, transgenic 129S6/Tg2576 mice spent 32% less time in the target quadrant than WT mice (Fig 5D). The percent time spent in the annulus was 41% lower on day 14 (*p* = 0.038) and 45% lower on day 17 (*p* = 0.023) for 129S6/Tg2576 mice compared to WT mice (Fig 5E). The difference in search accuracy was also apparent in individual swim paths (S2 Fig) and the group occupancy plot (Fig 5F) showing a more distinct pattern for WT mice.

### Radial Arm Water Maze

The radial arm water maze test introduces an ‘episodic-like’ component to the spatial memory paradigm that is tested in the Morris water maze, and thus, is used to evaluate both reference and working memory simultaneously [29, 39]. For radial arm water maze testing, animals were trained for nine days followed by three days of testing. Fig 6 shows the data as average number of errors per trial.

Saline-injected control mice rapidly learned the new platform location, which significantly reduced their error rate from 8.0 ± 0.79 in trial 1 to 0.8 ± 0.18 in trial 4 (Fig 6A). With 1.0 ± 0.48 errors, saline-injected control mice also performed better in the retention trial (trial 5). In contrast, scopolamine-treated mice did not perform as well in trials 2–5, error rates ranged from 6.1 ± 0.95 to 8.2 ± 1.0. To evaluate long-term memory (24 h), we assessed the entries into the target arm of the previous test day for days 2–12. Saline-injected control mice had 48.6% more entries into the arm than scopolamine-treated mice (Fig 6B) indicating that scopolamine treatment impaired long-term memory.

Fig 6C shows that after the learning phase, 129S6/Tg2576 mice consistently had higher error rates than WT mice with error numbers significantly different on trial 3 (3.6 ± 0.4 vs 5.3 ± 0.4) and trial 4 (2.0 ± 0.3 vs. 3.9 ± 0.4). We did not observe statistical differences in performance between WT and transgenic Tg2576 mice in the retention trial (trial 5). 129S6/Tg2576 mice entered the previous target arm less often than WT mice (19.9 ± 0.9 vs. 16.6 ± 1; Fig 6D), but this difference was not statistically significant.

### Spontaneous Alternation (Y-maze)

Spontaneous alternation using a Y-maze is a test for habituation and spatial working memory [37, 40]. The animal is allowed to freely explore all three arms of the Y-maze and spontaneous alternation is calculated. We did not observe any significant differences in spontaneous alternation between scopolamine-induced dementia mice and control mice (Fig 7A). Overall, scopolamine-induced dementia mice had 58% more total arm entries than saline-injected control mice (*p* < 0.001) suggesting increased general activity produced by scopolamine as reported before (Fig 7B; [22, 41]). No difference in spontaneous alternation or the number of total arm entries was observed between WT and 129S6/Tg2576 mice (Fig 7C and 7D). Moreover, spontaneous alternation was comparable between all four groups of mice. All mice alternated between arms above chance level (22.2%), indicating that neither cohort showed impairment in this test.

## Discussion

Below we discuss the test battery we established and validated in the present study, as well as the data resulting from using this battery in two dementia mouse models on a 129S6 background.

### 129S6 Background Strain

Mouse strains vary in behavior and cognitive performance, and strain differences in activity, anxiety, and exploratory behavior are reflected in different learning patterns and cognitive performance [32–36]. Thus, one cannot assume that B6;SJL/Tg2576 and 129S6/Tg2576 mice have identical behavioral patterns and memory impairment. Despite the large numbers of studies that have been conducted using B6;SJL/Tg2576 mice, cognitive data from 129S6/Tg2576 mice are limited. To establish a comprehensive test battery, we first conducted tests in scopolamine-treated 129S6 mice. Scopolamine-induced dementia has been studied in rodents and humans, but no work has been published on scopolamine-induced dementia in 129S6 mice [42, 43]. We dosed 3-5-month old 129S6/Tg2576 mice with vehicle or 10 mg/kg scopolamine (i.p.) at the beginning of each test day, and then evaluated all mice of both groups in the test battery.

Original Tg2576 mice were bred on a B6;SJL mixed background (B6;SJL/Tg2576) and have been tested in many behavioral studies [5, 8–11, 31, 44, 45]. B6;SJL/Tg2576 mice have memory impairment starting at about 6 months of age [7], but in some studies memory impairment could not be reproduced [31, 44, 46]. Since B6;SJL/Tg2576 mice carry the recessive *Pde6brd1* (*rd1*) mutation, homozygous *rd1* carriers have retinal degeneration and visual deficits at 7–9 weeks of age [12, 15, 47]. Therefore, data obtained from cognitive tests that rely on visual cues and require integration of visuospatial information could be distorted and unreliable, which might explain some of the observations made in previous studies [15–17].

In contrast, Tg2576 mice on a 129S6 background (129S6/Tg2576) do not carry the *rd1* mutation causing visual deficits. Nevertheless, only few studies utilized 129S6/Tg2576 mice to evaluate memory decline [18–20, 48–50]. Compared to age-matched WT control mice, 6-month old 129S6/Tg2576 mice display cognitive impairment in the novel object recognition task and the Morris water maze [20]. Hongpaisan et al. [19] detected impaired spatial learning and memory in 5-month old 129S6/Tg2576 mice when tested in the Morris water maze. Rustay et al. [18] examined cognitive performance in 6-month old B6;SJL/Tg2576 mice and in 129S6/Tg2576 mice at 6, 12 and 18 months of age. In this study, both 6-month old B6;SJL/Tg2576 and 129S6/Tg2576 mice were impaired in a contextual fear conditioning test. However, behavior of B6;SJL/Tg2576 mice in a spontaneous alternation test was inconsistent, with only one of two tested groups showing a cognitive deficit. In contrast, both groups of 6 month-old 129S6/Tg2576 mice showed cognitive impairment in this task. In addition, Rustay et al. [18] showed that 7-month old 129S6/Tg2576 and B6;SJL/Tg2576 mice have comparable Aβ plasma concentrations and plaque loads in the brain. However, with fewer than ten published studies evaluating cognitive function in 129S6/Tg2576 mice, behavioral data on this strain remain limited [18–20, 48–50].

### Behavioral Test Battery

We considered four points when selecting the cognition assays for the test battery. First, we selected cognitive tests that evaluate several aspects of memory loss to account for deficits in learning memory (Morris and radial arm water maze), working memory (alternation), object discrimination (novel object recognition), spatial memory (Morris and radial arm water maze), long-term memory (radial arm water maze), and episodic-like memory (radial arm water maze) [24, 29, 35, 51–53]. Second, we selected tests that have been established for the original B6;SJL/Tg2576 mouse model [5, 8–11, 31, 44, 45]. Third, we designed the test battery for use in long-term studies assessing learning and memory of 129S6/Tg2576 mice, and therefore, considered impairments that could potentially occur in mice during aging (e.g., decreased muscular strength, motor coordination and balance; [54–56]). Given that 129S6 mice naturally have high anxiety and low activity levels, which can lead to poor performance in behavioral tests such as passive avoidance or the Barnes maze, we selected tests that are minimally invasive and stressful [34, 57–60]. Fourth, we omitted tests involving food restriction, since this can influence Aβ levels and cognitive performance in mouse AD models [61, 62].

All tests were conducted in order of increasing invasiveness, with exception of the Y-maze continuous alternation test. This test was conducted at the end of the test battery to minimize interference with the forced alternation test at the beginning of the battery (Fig 1). Locomotor activity data for the behavioral test battery are summarized in Table 1.

### Forced Alternation (Y-Maze)

In our experiments using the forced alternation test, we did not observe statistically significant differences between scopolamine-induced dementia mice and control animals (Fig 2A and 2B). This could be due to two phenomena: One possibility is that the mice did not recognize the previously visited arms, and thus, regarded all three arms as novel. To increase recognition and navigation in the Y-maze, the end of each arm was marked with a different black/white pattern and visual cues were installed in the testing room. However, the possibility remains that the mice might not have been able to discriminate between the arms.

A second explanation for the lack of novel arm preference might lie in the high anxiety-like behavior of the 129S6 mouse strain [34, 57–59, 63]. Overall, mice in this study showed between 22 and 35 arm entries over the course of 5 min, which is low compared to literature values of other mouse strains, and might indicate anxiety [33, 34, 63, 64].

In contrast, we observed statistically significant differences in the time spent in the novel arm and in forced alternation between WT and 129S6/Tg2576 mice (Fig 2C and 2D). All WT mice entered the novel arm first when it was opened. Mice had in average less than 24 arm entries over the course of 5 minutes [33, 34, 63, 65].

### Novel Object Recognition

The results from our experiments with 10 mg/kg scopolamine indicate that this dose allows reliable and reproducible induction of dementia in the 129S6 mouse strain. This observation is consistent with reports from other groups that used 0.1–10 mg/kg scopolamine for the novel object recognition test in other mouse strains [66–68]. Our data show that saline-injected control mice interacted more with the novel object compared to scopolamine-treated mice (Fig 3A, *p* = 0.47). Although there is a clear trend, this effect was not statistically significant. In fact, overall object interaction for 129S6 control mice was only about 50% for both objects indicating that potential cognitive impairment of scopolamine-treated mice could have been masked by low object interaction of the control mice. 129S6/Tg2576 mice at 11–13 months of age showed a trend to interact less with the novel object, but this trend was also not statistically significant (*p* = 0.257). Overall, we expected the observed trends to be larger since it has been shown that object recognition is significantly impaired in 6-12-month old 129S6/Tg2576 mice when compared with age-matched non-transgenic control mice [20]. Several reasons might account for the small effects seen in the novel object test.

First, the length of the intertrial interval determines the level of difficulty of this memory task, and thus, the performance in the object recognition test [26, 68, 69]. Indeed, an extended intertrial interval can lead to low performance [25, 68]. In the present novel object recognition test protocol, we incorporated a one-hour intertrial interval, which is a common intertrial interval in protocols of scopolamine-induced dementia and the Tg2576 model [20, 26, 66, 69, 70]. However, it is possible that a shorter intertrial interval could have resulted in a better performance of the control group.

Second, 129S6 mice have high baseline anxiety-like behavior, which could explain the lack of interaction with the novel object, and thus, performance at chance level as we observed in all groups [33, 34]. Therefore, the little object interaction could be in part due to the specific genetic background of the mice tested [33, 34, 57–59, 63].

Third, another potential reason why the trends were not statistically significant could be due to the similarity of the objects used in this test. In general, object interaction increases with increasing complexity of the object and the two objects have to be different enough for mice to distinguish between them [25]. The objects used in our tests—a cube and a sphere—were simple in shape and similar in appearance (same material and color, and similar size). We used these objects because in preliminary experiments mice showed no object preference, whereas object preference was observed when we used more complex objects. Nevertheless, conducting the novel object recognition test with more complex, distinguishable objects might have resulted in better recognition of the novel object. Therefore, we repeated this test with 4-month old Tg2576 mice (n = 6) and WT mice (n = 8) using different and more distinct objects (S3A Fig). Compared to sample object interactions, we found significantly increased interaction with the novel object (*p* = 0.02) in WT mice, but not in Tg2576 mice. These results suggest that the performance of WT and Tg2575 mice in this test depends on the animals’ ability to discriminate between objects. Therefore, we suggest using more distinct objects for future tests.

Together, detecting memory deficits in the novel object recognition task clearly depends on several factors (i.e., experimental protocol, objects, background strain, etc.), and low performance of the 129S6 control mice and the WT mice in the novel object task observed in this study could also be a combination of the factors mentioned above.

### Morris Water Maze

It has previously been demonstrated in the Morris water maze that scopolamine dosing induced impairment [66, 71]. Zhang et al. [71] showed that Kunming mice dosed with 0.5 or 1 mg/kg scopolamine (i.p.) had increased latency times in the spatial acquisition phase. Han et al. [66] showed similar learning and memory deficits after treatment with 0.3 or 0.6 mg/kg scopolamine. In both studies, mice had dose-dependent cognitive impairment in the retention trial. Here we show that treating mice with scopolamine impaired both learning in the cued learning phase and memory in the spatial acquisition and retention phases (Figs 4 and 5).

Cognitive impairment of B6;SJL/Tg2576 mice in the Morris water maze was first demonstrated by Hsiao et al. [5]. This group reported impairment in cued learning, spatial acquisition, and retention trials in 9-10-month old B6;SJL/Tg2576 mice, but not in 2-3- or 6-month old B6;SJL/Tg2576 mice [5]. Since then, multiple studies have been conducted by this and other groups using B6;SJL/Tg2576 mice in the Morris water maze [7, 9, 28, 31]. However, retinal degeneration can confound experimental data in this test and introduce an impediment if outcomes are analyzed without considering the *rd* genotype [16, 17]. Therefore, a visual cue is sometimes included in the cued learning phase to verify that mice have adequate vision [72]. In contrast, 129S6/Tg2576 mice that have no visual defect are a reliable alternative for behavioral studies [18]. A recent study by Ohta et al. [20] showed increased spatial acquisition latencies in 129S6/Tg2576 mice at 6 and 12 months of age. Our own data show that 129S6/Tg2576 mice were impaired during the late stages of the Morris water maze, although the differences were less prominent in the experiments involving mice with scopolamine-induced dementia (Figs 4 and 5).

### Radial Arm Water Maze

The radial arm water maze test is used to assess episodic-like memory in rodents, which is one of the earliest clinical symptoms of AD [29, 39, 73, 74]. In 2005, Savonenko et al. [29] used a test battery including the radial arm water maze and showed age-dependent impairment of episodic-like memory, which preceded impairment of reference memory in the double-transgenic APPswe/PS1dE9 AD mouse model.

One report by Wolff et al. demonstrated memory deficits in the scopolamine-induced dementia model with 129/Sv mice using the radial water maze test [75]. Scopolamine treatment (0.8 mg/kg; i.p.) did not influence performance at a 5 min inter-trial interval, but impaired performance at a 60 min inter-trial interval [75]. In our study, however, scopolamine-injected 129S6 mice showed significant deficits in both episodic-like memory (10 min inter-trial interval) and long-term memory (24 h; Fig 6A and 6B).

Some studies have tested Tg2576 mice in the radial arm water maze [10, 45, 76–80]. In a study with C57/Tg2576 mice, Morgan et al. [77] found no cognitive deficit in mice at 11½ months of age, but demonstrated cognitive decline in 15½-month old mice. The study utilized a protocol to train mice to ‘asymptotic performance’ in a 6-arm radial water maze. In another study, Morgan et al. [45] utilized a novel 2-day protocol and showed that 8-month old C57/Tg2576 mice had memory deficiencies. Arendash et al. [81] showed impaired memory in Tg(APPsw) mice at 8–9 months of age. In a recent study, Kiyota et al. [76] showed cognitive deficits in 129-Sve/Tg2576 mice at 8–9 months of age using a 6-arm radial arm water maze, whereas mice at 2–3 months of age had no such deficits. While these studies used different protocols, they all showed cognitive impairment in transgenic mice on different background strains at age 8 months and older. However, our study is the first report on the performance of 129S6/Tg2576 mice in the radial arm water maze task. We show that in the learning phase of the radial arm water maze test, the performance of 129S6/Tg2576 mice was significantly impaired (Fig 6C), but no difference in long-term memory was observed between WT and 129S6/Tg2576 mice (Fig 6D).

### Spontaneous Alternation (Y-Maze)

In the spontaneous alternation test, mice explored the same Y-Maze used for the forced alternation test. We did not find differences in spontaneous alternation behavior between control and scopolamine-treated mice, but observed more arm entries for scopolamine-treated mice (Fig 7A and 7C). 129S6/Tg2576 mice also did not show a difference in spontaneous alternation compared to WT animals (Fig 7B and 7D).

In contrast, decreased levels of spontaneous alternation were shown in Swiss-Webster mice dosed with 1 mg/kg scopolamine-HBr and in A/J, DBA/2J and C57BL/6J mice dosed with 10 mg/kg scopolamine-HBr compared to saline-injected mice [22, 82]. Rustay et al. [18] reported decreased spontaneous alternation in 129S6/Tg2576 mice at 6 and 12 months of age, but not at 18 months of age. Overall, the available data from Tg2576 mice on a B6;SJL or a mixed C57 background vary widely, with some studies showing memory deficits as early as 3 months of age [28, 83], while others detect no difference in mice at various ages [17, 28, 44–46]. Our data in combination with literature findings suggest that the spontaneous alternation test does not reliably detect cognitive impairment in scopolamine-treated 129S6 or 129S6/Tg2576 mice, and therefore, should not be a stand-alone test to evaluate memory impairment in mice on a 129S6 background.

## Conclusions

To the best of our knowledge, this is the first study utilizing a comprehensive behavioral test battery to evaluate working memory, object discrimination, spatial memory, learning and episodic-like memory in two dementia mouse models on a 129S6 background. The test battery we used consists of five non-invasive behavioral tests (forced alternation (Y-maze), novel object recognition, Morris water maze, radial arm water maze, and spontaneous alternation test (Y-maze)).

Both, scopolamine-injected 3-5-month old 129S6/SvEvTac mice and 11-13-month old transgenic 129S6/Tg2576 mice revealed memory deficits compared to their respective controls. However, these models exhibited cognitive impairment in varying patterns. Scopolamine-treated 129S6/SvEvTac mice had impaired spatial learning and memory, as well as impaired episodic-like learning, retention and long-term memory, but had no effects in cognitive performance in the novel object recognition or in alternation tasks.

On the other hand, 129S6/Tg2576 mice had impaired spatial memory and spatial and episodic-like learning, but retention and long-term memory in the radial arm water maze task were not impaired. Similar to scopolamine-treated mice, 129S6/Tg2576 mice did not exhibit significant cognitive deficits in the novel object recognition or the spontaneous alternation task. Thus, these behavioral tests may be of limited use for the 129S6 background strain. However, working memory (forced alternation test) and long-term memory (Morris water maze, radial arm water maze) were significantly impaired in 129S6/Tg2576 mice.

Together, the test battery introduced here allows for reliable detection of cognitive impairment in mice on a 129S6 background and the data obtained are comparable to those from other background strains. Due to the modular nature of this test battery, more behavioral tests, e.g., invasive assays to gain additional cognitive information, can easily be added.

## Supporting Information

S1 Fig Morris Water Maze Individual Swim Paths for Scopolamine-Demented Mice. S1 Fig shows individual swim paths for control and scopolamine-injected mice during the retention trial on day 17 of the Morris water maze task.
(TIFF)

S2 Fig Morris Water Maze Individual Swim Paths for 129S6/Tg2576 Mice. S2 Fig shows individual swim paths for wild-type and 129S6/Tg2576 mice during the retention trial on day 17 of the Morris water maze task.
(TIFF)

S3 Fig Novel Object Recognition Test in 4-month old WT and Tg2576 mice. A) Sample object (left; 50 ml Falcon^®^ tissue culture flask filled with sand, 9.5 cm high, 2.5 cm deep, 5.5 cm wide) and novel object (right; DUPLO bricks, 8-cm high and 3.2-cm wide) used for this test. B) Object interaction shown in % for WT (n = 8) and Tg2576 mice (n = 6). WT mice showed significant preference (*p* = 0.02) for the novel object over the familiar object. Data are mean ± SEM. Mice did not have a natural preference for either object (data not shown).
(TIFF)

## Abbreviations

AD: Alzheimer’s disease
Aβ: amyloid beta
hAPP: human amyloid precursor protein
