# Supplementary figures and images for: A Comprehensive Behavioral Test Battery to Assess Learning and Memory in 129S6/Tg2576 Mice
Source: PLoS One. 2016 Jan 25;11(1):e0147733. doi: 10.1371/journal.pone.0147733 (PMC4726499; doi:10.1371/journal.pone.0147733)

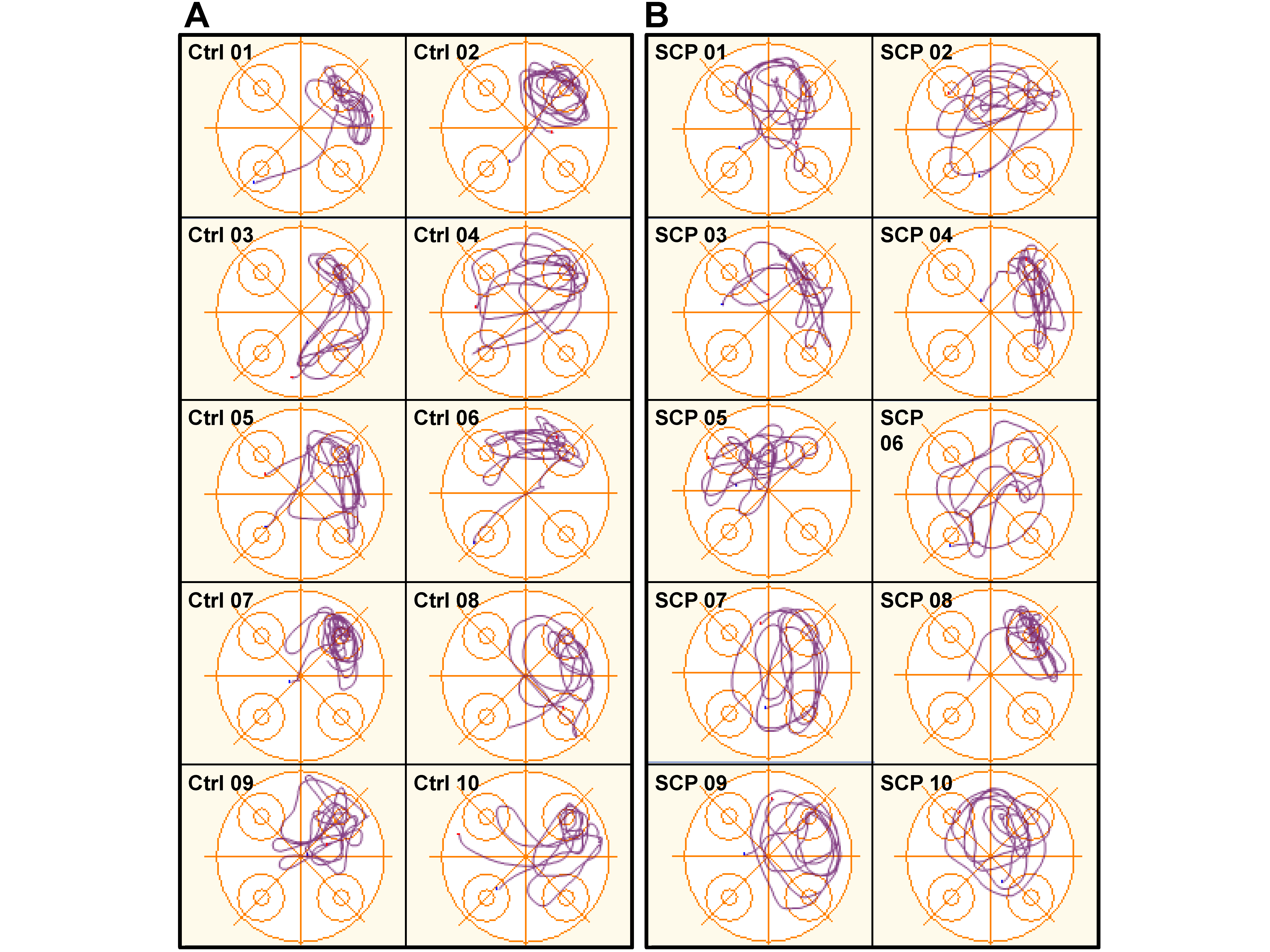

Supplement: S1 Fig — shows individual swim paths for control and scopolamine-injected mice during the retention trial on day 17 of the Morris water maze task. (TIFF) [file pone.0147733.s001.tiff]

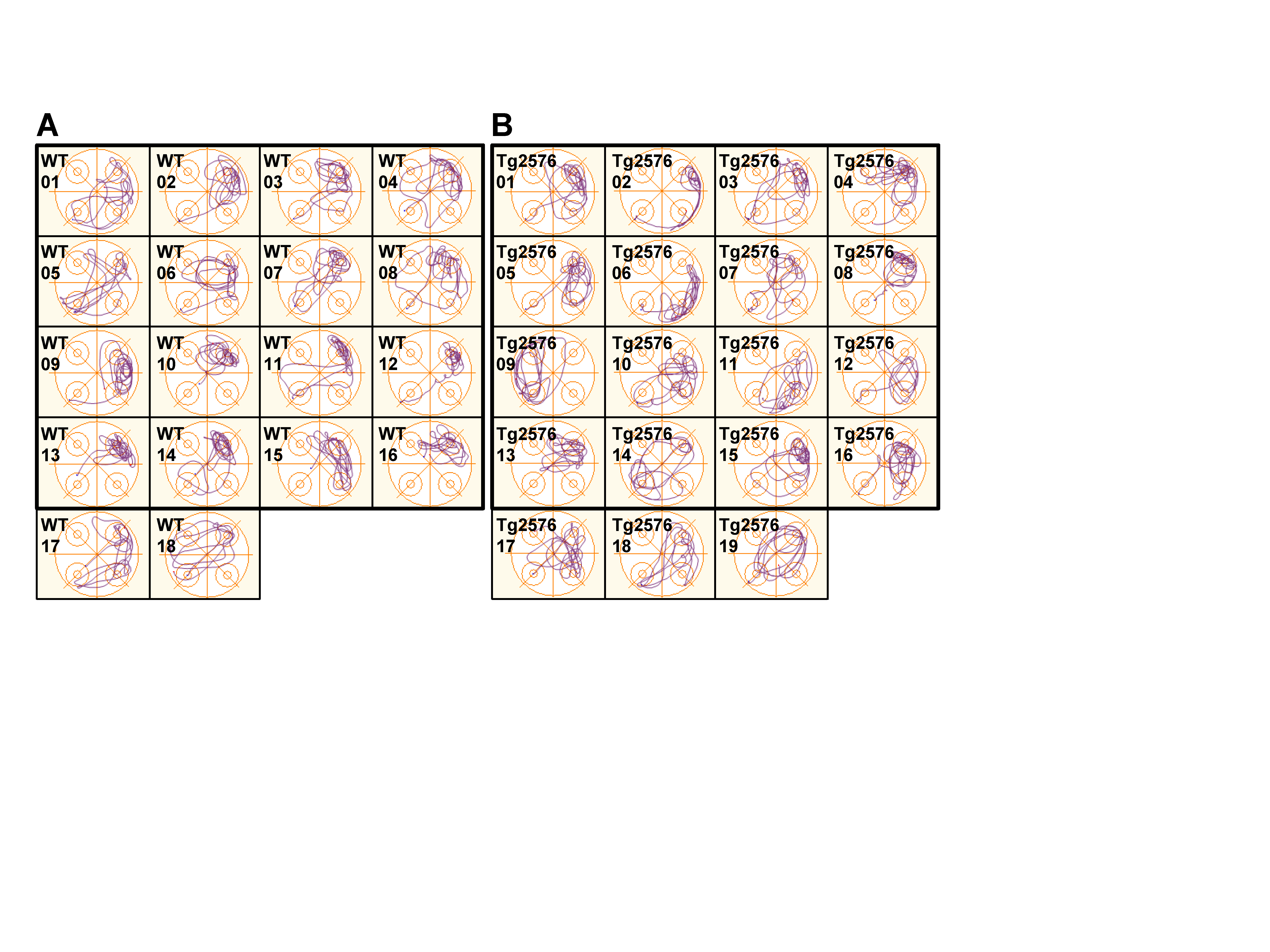

Supplement: S2 Fig — shows individual swim paths for wild-type and 129S6/Tg2576 mice during the retention trial on day 17 of the Morris water maze task. (TIFF) [file pone.0147733.s002.tiff]

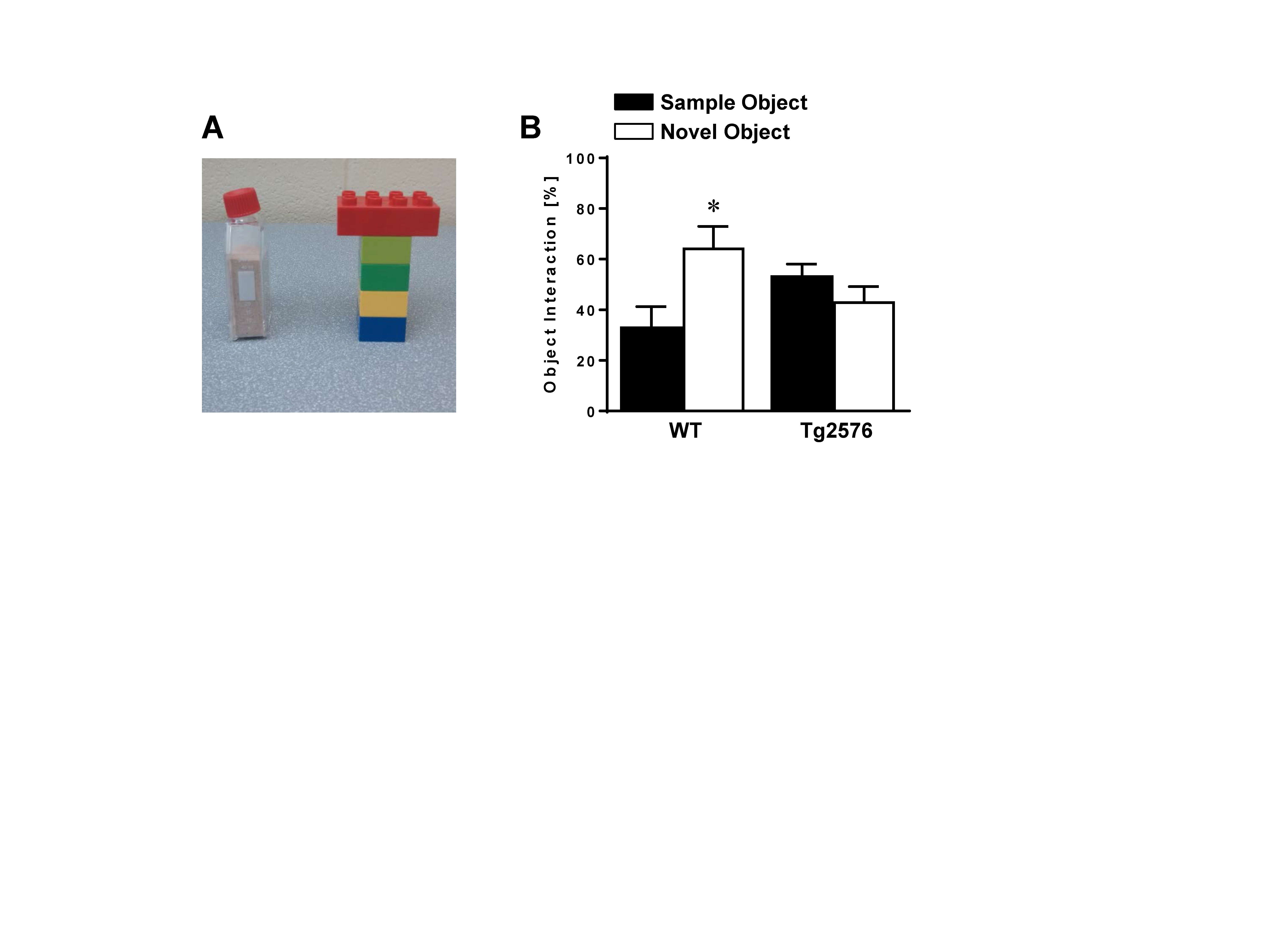

Supplement: S3 Fig — A) Sample object (left; 50 ml Falcon® tissue culture flask filled with sand, 9.5 cm high, 2.5 cm deep, 5.5 cm wide) and novel object (right; DUPLO bricks, 8-cm high and 3.2-cm wide) used for this test. B) Object interaction shown in % for WT (n = 8) and Tg2576 mice (n = 6). WT mice showed significant preference (p = 0.02) for the novel object over the familiar object. Data are mean ± SEM. Mice did not have a natural preference for either object (data not shown). (TIFF) [file pone.0147733.s003.tiff]
